# Foreword

**DOI:** 10.1080/17482631.2017.1319588

**Published:** 2017-05-22

**Authors:** Robert Whitaker

If we were to turn the ADHD clock back to the 1980s, we would find a story that, from a global perspective, could perhaps be titled Those crazy Americans: There they go again.

The diagnosis back then was brand new, one that the American Psychiatric Association had created in 1980 when it published *DSM III*, and for the next 15 years or so the use of this diagnosis and prescribing of stimulants to school-age youth was mostly an American affair, looked upon with considerable skepticism by psychiatrists and physicians in Europe and other parts of the world. In 1995 the US Drug Enforcement Agency reported that the US “produces and consumes five times more methylphenidate than the rest of the world combined”, which the agency saw as a rather dubious achievement.^1^

And so it seemed 25 years ago: psychiatrists and physicians in other countries weren’t about to follow this latest American practice and start seeing their children through the ADHD lens. Perhaps, I remember thinking, the US medical community could learn from the rest of the world, and this would put a brake on the ADHD train.

But here we are in 2017, and ADHD has become a global disorder: 5% of school-age children are said to “have” ADHD, and the prescribing of stimulants to children and youth has risen in countries around the world. This globalization effort has marched forward under the biological banner. The condition is said to be highly heritable (and thus a genetic disorder), and brain scans tell of how brain volumes differ in ADHD children compared to “normal” youth. Thus we have an important question to ask: Is the ADHD phenomenon a story of scientific progress, or the successful exportation of an American fad?

To raise this question is not to say that there are not children who have behavioral problems characterized by inattention or “hyperactivity” or that there are not children who are floundering in school and need help. The question is whether societal beliefs about ADHD are grounded in a good understanding of the relevant science and whether the use of the diagnosis and the prescribing of medications has proven helpful to children who may be struggling in this way.

This issue of *The International Journal of Qualitative Studies on Health and Well-being* is devoted to “challenging the ADHD consensus”, which provides a much-needed forum for critiques of the common wisdom. The first thing that readers may notice is that the eight articles reflect the globalization of ADHD. They tell of media coverage of ADHD in France and Australia; of youth in Chile and European countries so diagnosed; and of academic researchers in the UK, the Netherlands and elsewhere investigating the phenomenon.

The globalization of ADHD relies on the telling of a story to the public, and in an analysis of media reports in France, a society usually portrayed as resistant to this “medicalization of childhood”, Ponnou and Gonon report that whereas newspapers and other print media have often provided fairly nuanced coverage of ADHD, giving space to psychosocial explanations for ADHD behaviors, television shows—where most Europeans get their news these days—have mostly presented the biomedical narrative. When such programs were aired, parental requests for the ADHD diagnosis rose.

Meanwhile, Harwood and her research colleagues’ study of the selling of ADHD in Australia reveal how media reports of ADHD employed the language of “heroic struggles, criminals and scientific breakthrough”. Science was on the march; the medication provided a “lubricant for learning” and yet parents, in their desperate bid to find help for their children, had to fight against “bad” doctors who refused to take the ADHD diagnosis seriously. This was a framework that told of a valiant fight for good, and with such stories forming the public understanding of ADHD, the prescribing of stimulants to children in Australia, at least for a time, surpassed the rate in the original ADHD hotspot, the USA.

The globalization of ADHD has also involved the “educating” of professionals—teachers, nurses, physicians, and other mental health workers—and the article of Meerman and co-authors documents how this instruction might best be described as scientifically illiterate. To cite just one example, the biomedical narrative tells of how brain scans show an abnormality in brain volumes in ADHD children. But of course such research involves calculating “average” brain volumes from youth diagnosed with ADHD compared to “normal” controls, which means that a scatterplot of individual brain volumes would show many in the ADHD cohort as “perfectly normal” and many in the “normal” cohort with abnormally “small” volumes.

The fact that “most children with ADHD behavior have ‘normal’ brains” is one of six facts that Meerman, Batstra, Grietens, and Frances argue that teachers and other mental health professionals “need to know”. The remarkable thing is that so many mental health professionals would be surprised to learn of this basic information.

One fact that is noted in several of the articles, and is almost unbearably sad, is that studies have regularly failed to find that ADHD medications provide a long-term benefit. The studies have not found that the drug treatment reduces delinquency rates, or improves school performance, or improves any other markers of success. In the absence of such benefits, we are left chalking up the long-term harms that can come with regular stimulant use. Regardless of whether ADHD is “real” or simply a diagnostic construct, we should want to see that the treatment helps school-age children grow up and thrive. Given that research hasn’t shown that to be so, what is the rationale for long-term stimulant use?

This question is of particular relevance given that we known there are psychosocial interventions that can help such children. Timimi tells of the success of a Relational Awareness Programme that involves “prioritizing building relationships over controlling behavior (symptoms)” and helps parents and teachers move “away from emotional energy reinforcing negatives and toward reinforcing positives”. My guess is that if you asked 100 random adults in society to assess the likely effectiveness of these two therapies, stimulants or fostering relationships, that helped build a child’s emotional resilience, nearly all would pick the latter. But the globalization of ADHD has not proceeded with that menu of options put before society.

In debates over ADHD, we often fail to hear from the children so diagnosed and medicated. What does it do to their inner world? How does the diagnosis affect their sense of self? How do they navigate their new status in the world as an ADHD child? Rojas Navarro and Vrecko, Helle-Valle et al., and Salomonsson tell of investigations into the child’s world, and given the stereotype of ADHD children as difficult and prone to outbursts, these articles all remind us, in one way or another, of how resilient, caring, and frightened they may be. Which is to say, they remind us of children struggling to make their way in a challenging world, who could use help of a non-medical kind.

*Journalist and author of* Anatomy of an Epidemic *and founder of Madinamerica.com*

## Note

1. US Department of Justice, Drug Enforcement Administration. (1995). Methylphenidate: A Background Paper.

